# Using machine learning for image-based analysis of sweetpotato root sensory attributes

**DOI:** 10.1016/j.atech.2023.100291

**Published:** 2023-10

**Authors:** Joyce Nakatumba-Nabende, Claire Babirye, Jeremy Francis Tusubira, Henry Mutegeki, Ann Lisa Nabiryo, Sudi Murindanyi, Andrew Katumba, Judith Nantongo, Edwin Sserunkuma, Mariam Nakitto, Reuben Ssali, Godwill Makunde, Mukani Moyo, Hugo Campos

**Affiliations:** a Department of Computer Science, Makerere University, Uganda; b Makerere Artificial Intelligence Lab, Makerere University, Uganda; c Marconi Machine Learning Lab, Makerere University, Uganda; d Department of Electrical and Computer Engineering, Makerere University, Uganda; e International Potato Center (CIP), Uganda; f International Potato Center (CIP), Mozambique; g International Potato Center (CIP), Kenya; h International Potato Center (CIP), Peru

**Keywords:** Sweetpotato, Flesh-colour, Mealiness, Image analysis, Machine learning

## Abstract

The sweetpotato breeding process involves assessing different phenotypic traits, such as the sensory attributes, to decide which varieties to progress to the next stage during the breeding cycle. Sensory attributes like appearance, taste, colour and mealiness are important for consumer acceptability and adoption of new varieties. Therefore, measuring these sensory attributes is critical to inform the selection of varieties during breeding. Current methods using a trained human panel enable screening of different sweetpotato sensory attributes. Despite this, such methods are costly and time-consuming, leading to low throughput, which remains the biggest challenge for breeders.

In this paper, we describe an approach to apply machine learning techniques with image-based analysis to predict flesh-colour and mealiness sweetpotato sensory attributes. The developed models can be used as high-throughput methods to augment existing approaches for the evaluation of flesh-colour and mealiness for different sweetpotato varieties. The work involved capturing images of boiled sweetpotato cross-sections using the DigiEye imaging system, data pre-processing for background elimination and feature extraction to develop machine learning models to predict the flesh-colour and mealiness sensory attributes of different sweetpotato varieties. For flesh-colour the trained Linear Regression and Random Forest Regression models attained $R^{2}$ values of 0.92 and 0.87, respectively, against the ground truth values given by a human sensory panel. In contrast, the Random Forest Regressor and Gradient Boosting model attained $R^{2}$ values of 0.85 and 0.80, respectively, for the prediction of mealiness. The performance of the models matched the desirable $R^{2}$ threshold of 0.80 for acceptable comparability to the human sensory panel showing that this approach can be used for the prediction of these attributes with high accuracy. The machine learning models were deployed and tested by the sweetpotato breeding team at the International Potato Center in Uganda. This solution can automate and increase throughput for analysing flesh-colour and mealiness sweetpotato sensory attributes. Using machine learning tools for analysis can inform and quicken the selection of promising varieties that can be progressed for participatory evaluation during breeding cycles and potentially lead to increased chances of adoption of the varieties by consumers.

## 1 Introduction

Sweetpotato (Ipomoea batatas L. Lam) is a food security crop in sub-Saharan Africa whose significance has grown due to the major threats to bananas and cassava caused by pests and diseases [1], [2]. In Uganda, sweetpotato is ranked third after cassava and bananas as the most-consumed staple crop and is considered a significant source of livelihood for smallholder farmers [3] and nutritious food [4]. Sweetpotato breeding programs have developed new varieties with increased resistance to several environmental stress factors, pests, and diseases and satisfy consumer needs [5], [1]. During the sweetpotato breeding process, breeders assess different phenotypic traits, such as the sensory attributes, to decide which varieties to progress to the next stage along the breeding cycle [5], [6]. Sensory attributes such as taste, smell, flavour and appearances like and texture, are important for consumer acceptability [7], [8], [9], [10]. These refer to characteristics of an agricultural product such as food that can be perceived through senses [11].

Colour of the food, for example, is the first parameter of quality evaluated by consumers. Flesh-colour and appearance are described as morphological traits that consumers typically use as visual cues to make decisions about the underlying quality attributes such as taste, cooking quality and texture of a particular crop. Mealiness has also been suggested as a priority characteristic in boiled roots [12]. Previous studies have shown that sensory attributes greatly influence consumer preference for crops like coffee, vegetables and sweetpotatoes [13], [14], [15], [16]. However, consumer acceptance remains a big challenge to adopting new sweetpotato varieties in Uganda [17]. Therefore, sensory attributes must be accurately measured during the breeding process. Sensory profiling of sweetpotato involves trained persons who rate sweetpotato samples for several defined sensory attributes [8], [11], [18]. In Uganda, food scientists have developed a lexicon and protocols for conducting descriptive sensory analysis of sweetpotato samples in a breeding program [16]. The measurement of sweetpotato sensory attributes through descriptive sensory analysis is essential because this gives the closest approximation to what consumers perceive. Visual appearance and mealiness are among the key driving attributes for sweetpotatoes acceptance, and enhancing these traits is a target for the breeding program. Previous work has also shown that contemporary breeding programs that often focused on agronomy-related characteristics such as yield and less on end-user preferences resulted in slow adoption of improved varieties [12]. This validates the need to consider sensory attributes that are user-preference centred during the breeding process.

Despite the research done over time, evaluating sweetpotato sensory attributes remains a challenge, mainly because it is a labour-intensive process where only a few samples can be evaluated at a time [18], [16]. Yet, breeding programs are tasked with screening several genotypes each harvest season. This makes using sensory panels costly regarding time and resources and unsuitable for early breeding stage trials where hundreds or thousands of genotypes must be evaluated. This challenge can be addressed by developing high-throughput methods that can be used to measure sweetpotato sensory attributes during the breeding process. Such methods include instrumental texture analysis, for instance, texture parameters using the wedge fracture test. Moreover, texture profile analysis protocols have been developed and are shown to correlate with various texture attributes of boiled sweetpotatoes [19], [20] particularly mealiness. Another example is the use of Hyper Spectral Imaging, which was shown to predict the optimal cooking time of boiled sweetpotatoes [21]. Near-Infrared Reflectance Spectroscopy (NIRS) technology has also been used to predict macro and micro-nutrients in root and tuber crops [22], [23], [24], [25]. The use of NIRS in variety screening can increase the throughput of analysis but this requires the use of specialised equipment [19].

Computer vision (CV) and machine learning (ML) have been used as techniques to perform image analysis in agriculture [26], [27]. These techniques enable the automatic and efficient extraction of useful information and patterns from large amounts of image data. Particularly, ML-based image analysis has been applied in agriculture for crop mapping [28], [29], [30], yield prediction [31], [32] and pest and disease detection [33], [34], [35], [36], [37], [38], [39], [40]. These techniques have given farmers and researchers valuable precision agriculture insights to help maximise crop yields and enhance decision-making throughout the crop production processes. Machine learning for sensory attribute prediction can significantly increase the throughput and accuracy of sweetpotato phenotype screening through rigorous model training on vast amounts of data. Research has shown that it is possible to use machine learning techniques combined with computer imaging to effectively predict sweetpotato sensory responses using the physical, chemical, and physical–chemical data [41], [42]. In [43], [44], [45], [46], hyper-spectral imaging was used to capture sweetpotato features which were then used to develop prediction models for various sweetpotato attributes such as colour, hardness, chewiness and moisture content using models like partial least square regression, least square support vector machines and multivariate linear regression. A major drawback with these approaches is that they rely on features from hyper-spectral imaging which requires specialized equipment to capture, compared to using the Red, Green, Blue (RGB) images. There is also limited work done to predict sweetpotato mealiness with machine learning and image-based analysis.

In this paper, we describe an approach to apply machine learning techniques with image-based analysis to predict flesh-colour and mealiness sweetpotato sensory attributes based on image data. The developed models can be used as a high-throughput method to augment existing approaches for evaluating flesh-colour and mealiness during breeding. This study seeks to answer the following research questions (RQs):

• *RQ1: What are the most important image features that contribute to flesh-colour and mealiness sweetpotato sensory attributes?*
• *RQ2: How do different machine learning algorithms perform in predicting flesh-colour and mealiness sweetpotato sensory attributes?*
• *RQ3: How do machine learning model results for flesh-colour and mealiness sweetpotato sensory attributes compare to the trained sensory flesh-colour and mealiness scores?*
• *RQ4: Can we develop a tool for breeders to predict flesh-colour and mealiness for sweetpotato samples?*

The main contribution of the paper is that we have shown that it is possible to use machine learning for image-based analysis of flesh-colour and mealiness sweetpotato sensory attributes. Our results also show that using machine learning techniques we can provide an acceptable level of accuracy for the flesh-colour and mealiness scores as compared to the ground truth scores from the trained sensory panel.

The rest of the paper is organized as follows: Section 2 discusses the methodology used in this paper. Section 3 discusses the flesh-colour and mealiness model results and evaluation. Section 4 discusses the process taken in model deployment and evaluation by the agricultural experts. Section 5 discusses the model, evaluation results and limitations. We conclude the paper in section 6.

## 2 Methodology

**Fig. 1:**
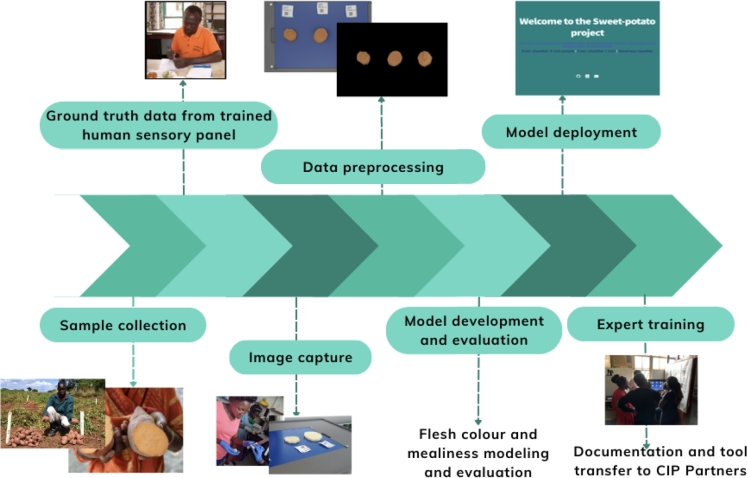
Approach taken in this research. The first step comprised of sweetpotato sample collection, which was followed with the collection of the ground truth data from the human sensory panel. The next step was the image data capture of boiled sweetpotato samples using the DigiEye machine, which was followed with the feature extraction step. The next steps included the development of the flesh-colour and mealiness models, model deployment, and model evaluation. The final step was expert training on using developed flesh colour and mealiness models.

Fig. 1 shows the approach and steps taken in this research. In the rest of this section, we describe these steps in detail.

### 2.1 Sample data collection

**Fig. 2:**
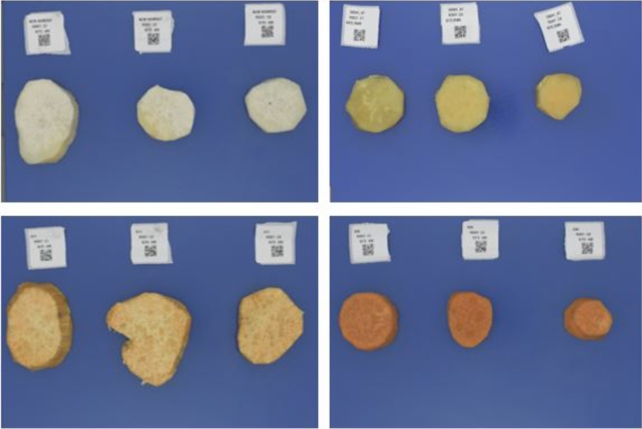
(a) White-fleshed sweetpotato boiled image samples. (b) Yellow-fleshed sweetpotato boiled image samples. (c)Orange-fleshed boiled sweetpotato image samples. (d) Deep Orange-fleshed boiled sweetpotato image samples.

As a first step, sweetpotato roots of various genotypes were harvested across different harvest trials in Uganda. This exercise was led by the sweetpotato breeding team at the International Potato Center (CIP) in Uganda. 217 sweetpotato phenotypes were collected between 2021 and 2022 [23]. The sweetpotato root samples selected for image-based data collection were obtained after the harvest. As shown in Fig. 2, the image data had variations in flesh-colour ranging from *white*, *yellow*, *orange*, and *deep-orange*. The harvested sweetpotato samples were cleaned and prepared according to the steps described in the RTBfoods Standard Operating Procedure for sample preparation using an established data collection protocol [47]. After the sample collection phase, the next step involved obtaining ground truth data and image data capture of the sweetpotato samples.

### 2.2 Ground truth data

In this research, we also relied on scores given for sensory attributes of the sweetpotatoes by a trained sensory panel following protocols described in [16]. We considered these scores as the ground truth data for our machine learning models. The trained human sensory panel is a panel that consists of 10-13 participants that rated boiled sweetpotato samples across different genotypes for different sensory attributes. The sweetpotato samples are steamed following a standard protocol [48] and served to the panel for evaluation. To establish the sensory profiles, the panel evaluated aroma and flavour attributes assessed by mouth, appearance attributes assessed by sight, and texture attributes assessed by mouth feel and by hand. We focused on flesh-colour and mealiness sensory attributes for boiled sweetpotato roots since these attributes are user-preference centered and drive the breeding selection of sweetpotatoes [1].

The human sensory panel assessed and scored the flesh-colour and mealiness sensory attributes among other aroma and flavour attributes [16]. Table 1 shows a sample of the human sensory panel results for flesh-colour, mealiness and positive peak force measured by the instrumental texture analysis of boiled sweetpotato roots to mimic mastication as described in [16]. The aggregated score for each attribute is a mean value given by the summation of all acceptable individual scores on the panel over the total number of experts. It was reported that it takes up to 30 minutes on average for a panelist on the sensory panel to provide sensory attribute scores for one sweet potato sample.

**Table 1:** Sample of the trained sensory panel results. The results presented here show the sweetpotato variety name and the mean values scored for flesh-colour intensity, mealiness by hand and positive force 1R sweetpotato sensory attributes.

Variety Name	Flesh-colour	Mealiness by hand	Positive force 1R
UGP20170334-27	0.4	8.2	8525
UGP20170335-6	1.5	4.4	6016
UGP20170028-7	3.9	5.9	4057
UGP20170015-26	1.4	7.0	4208
EJUMULA	8.1	2.0	4565
SILK OMUYAKA	0.3	7.9	8103
S36	6.7	4.8	3920
TANZANIA	2.6	8.0	6568
RESISTO CIP	9.0	0.7	1850
NASPOT 10	7.9	2.1	4630

The flesh-colour intensity attribute is visually assessed across the surface of the sample and scored according to a discrete scale of values ranging from **0-10** [16] where: **0** = white, **1** = cream, **3** = yellow, **5** = yellow-orange, **8** = orange and **10** = deep orange. The aggregated scores from the trained sensory panel are computed as mean values and used as ground truth data for label selection [16]. The mealiness by hand attribute is assessed by rubbing a portion of the sample between fingers and scored on a discrete scale of values ranging from **0-10** [16] according to the ease with which the sample breaks into small particles upon rubbing where: **0** = not mealy and **10** = extremely mealy. The peak positive force attribute is taken as a measurement of firmness - the higher the value, the firmer the sweetpotato sample. For each phenotype, three pieces of 3x3x2.5 cm are cut from each root and steamed for 35 minutes (from when the pot is placed on the gas fire). The texture of each piece at room temperature (20−25C∘) is analysed using a TA-XT texture analyzer [49] with a 10 kg load cell. The wedge fracture texture analysis determines the peak positive force (firmness).

The human sensory panel results for these attributes were used as ground truth data to validate the machine learning models we developed for flesh-colour and mealiness prediction. We obtained ground truth data for **227** samples harvested between October 2021 and November 2022.

### 2.3 Image capture

The next step after obtaining the boiled sweetpotato samples, was to capture the images of these samples. To do this, we used the DigiProduction imaging system, also known as DigiEye 700 mm Cube (VeriVide Limited, Leicester, UK), mounted with a D7500 Nikon DSLR camera (Nikon Cooperation, Tokyo, Japan) and 6500K system lights. The DigiEye machine is a 64-bit modular computer-controlled digital imaging system that comprises an illumination cabinet, a digital camera, a desktop personal computer and a monitor calibration, and a Digi-grade fastness assessment show results from flesh-colour [50]. Before using the DigiEye, several settings were required for accurate image capture as outlined in Table 2.

**Table 2:** Calibration settings for the different DigiEye components necessary for image capture.

DigiEye component	Calibration steps
Camera	Select camera installed on the machine
Illumination cabinet	10 minutes of warm up
Shutter speed	1/3
Camera aperture	8
White balancing	Use white board
Uniformity calibration	Use DigiEye digitizer chart

**Fig. 3:**
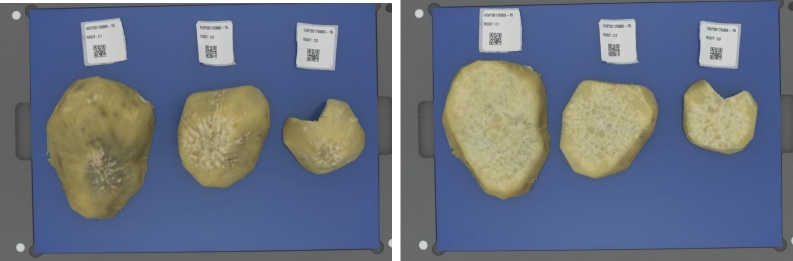
(a) Boiled After Peel sweetpotato sample. (b) Boiled Cross-section sweetpotato sample.

Following the settings and calibration steps in Table 2, we calibrated the DigiEye imaging system and obtained calibration values for RGB white colour space. The details of the calibration procedure are in [47]. At each step of image data capture, three root samples were collected for imaging. Each sample had a label with a QR code scanned for root details, for example, the specific phenotype and site where the samples were collected. The root samples and respective labels were placed on the DigiEye illumination cabinet to capture the images. Fig. 3 shows a sample of the captured sweetpotato root images. The samples are categorized into two different states:

1. **Boiled After Peel** - The raw after-peel sweetpotato samples are boiled after which the images are captured from the top.
2. **Boiled Cross-section** - The raw after-cross-section samples are boiled after which the images are captured.

The average time required from setup to the image capture was approximately 1 minute which was calculated from the total time taken to capture 120 images. A total of **1487** images of boiled sweetpotato samples were collected from **950** sweetpotato samples harvested between October 2021 to November 2022.

### 2.4 Data preprocessing

**Fig. 4:**
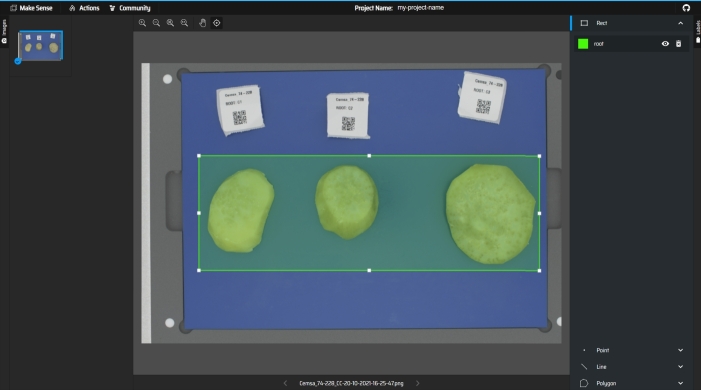
Example of image annotation process for the root detection model.

The next stage after image data capture was the development of the background extraction algorithm as part of the data preprocessing step. The algorithm was developed to distinguish the image background from the sweetpotato roots and concentrate the image analysis on the Region of Interest (ROI), which is the *root samples*. The background elimination process combined an object detection task followed by a clustering task. The object detection model was developed to identify the region with the roots within the image and improve the precision with which the clustering would be done. To develop the root detection model, a sample of **152** images with varying colour and size characteristics was annotated by drawing bounding boxes around the roots in the image as shown in Fig. 4. The image data annotation was done using the MakeSense^1^ annotation tool.

The annotated data was split into **91**, **38**, and **23** images for the training, validation and test sets, respectively. The root detection model was developed using YOLOv5 [51] a popular Convolutional Neural Network (CNN) used for object detection tasks [52], [53], [54]. For this task, a pre-trained YOLO model trained for object detection with the Common Objects in Context (COCO) dataset [55] was fine-tuned on the labelled sweetpotato dataset. Research has shown that by fine-tuning a pre-trained model, the YOLOv5 model can be trained much faster and attain high level of accuracy with even limited data compared to training a complete model from scratch for different types of tasks [56], [57], [58]. Specifically, a YOLOv5 small pre-trained model was used, and the training was done with the parameters specified in Table 3.

**Table 3:** Parameters for training YOLOv5 object detection model.

Batch Size	Epochs	Image size
32	120	640px

The fine-tuned object detection model attained a mean average precision (mAP) of 0.995 at 0.5 Intersection Over Union (IOU) on both the validation and test sets. This implies that the model had a very high accuracy for the task of identifying the region with the roots in the image.

**Fig. 5:**
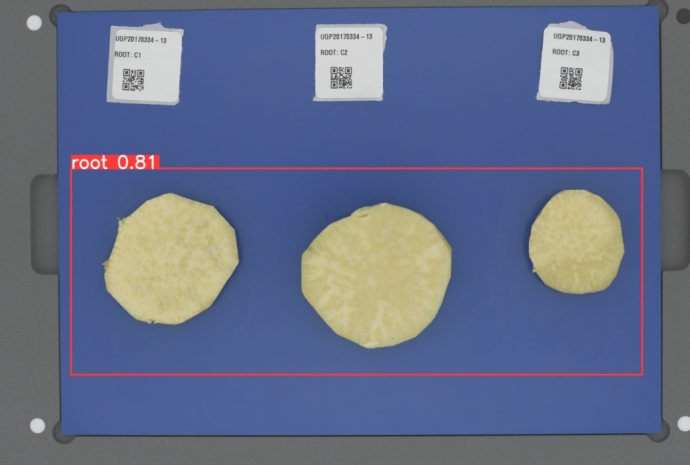
A sample result of root detection using the fine-tuned YOLOv5 model. The bounding box is used to determine the ROI in the image.

The next step in the background elimination was to develop a segmentation algorithm to get different parts of the image. Image segmentation classifies an image into different parts by grouping the image pixels. We used OpenCV's implementation of the K-means clustering algorithm [59] to group the image pixels into a defined number of clusters based on how close the pixel value intensities are to each other. The resultant clusters were then used to segment the image. The background elimination for an input image was done in two steps; in the first step, root detection with the fine-tuned YOLOv5 model was first applied to identify the region with roots in the image. One of the outputs of the root detection model is a bounding box around the roots and this information was used to select the ROI in the image as shown in Fig. 5.

**Fig. 6:**
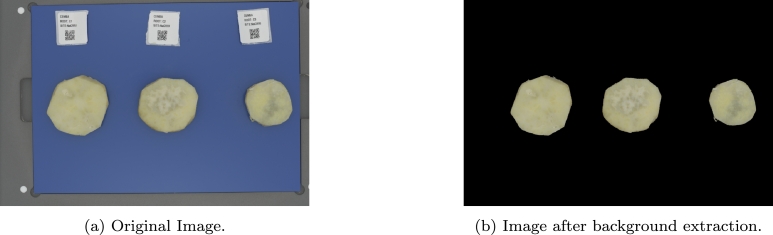
Original sweetpotato sample image captured from the DigiEye and a sweetpotato sample image after the background extraction.

In the second step, K-means clustering was applied to the ROI of the image for two clusters. A selection was then made based on the assumption that there were two distinct regions in the ROI representing the roots and the background. The clustering result grouped the pixels into two clusters representing the roots and the background. A mask was created to retain the pixels representing the root in the input image while the other pixels determined as the background was converted to black to segment the image. Fig. 6 shows the resultant image after applying the background extraction algorithm. The segmented images with the eliminated background were used as the image data input for the mealiness model development.

### 2.5 Exploratory data analysis for flesh-colour modelling

After the background extraction process, we carried out exploratory data analysis to understand flesh-colour variations across different sweetpotato samples. This enabled us to identify and select the relevant features for modelling. The results in this section were driven by answering the first research question.

**Fig. 7:**
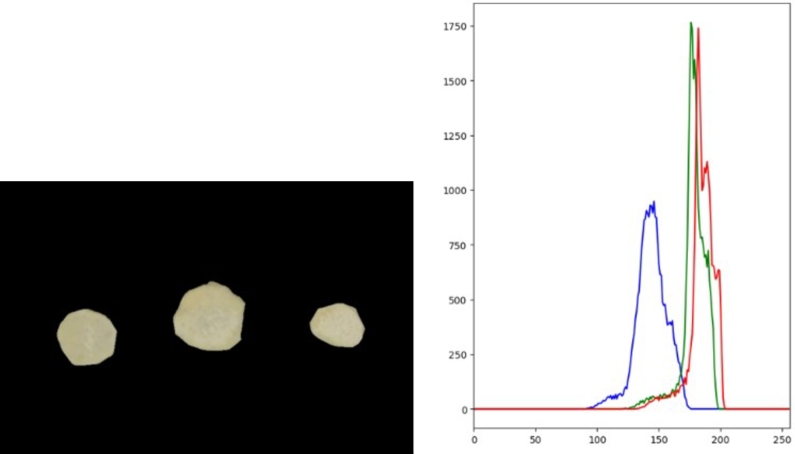
(L-R) White-fleshed samples and the pixel values represented on the RGB histogram.

**Fig. 8:**
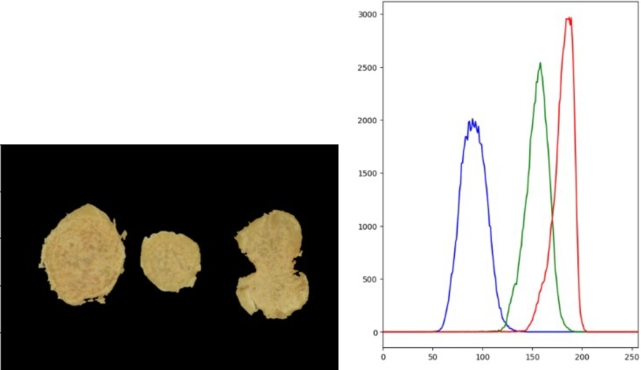
(L-R) Yellow-orange fleshed sweetpotato samples and the pixel values represented on the RGB histogram.

***RQ1: What are the most important image features that contribute to the sweetpotato flesh-colour and mealiness attributes?*** There were a number of attributes that were considered for flesh-colour modelling. Pixel intensity was one of the intrinsic attributes that was investigated for modelling. To visualize the pixel intensity distribution across the different image samples, pixel image data was extracted and analyzed on an RGB histogram. It was shown that different sweetpotato samples have distinct RGB bands, which is especially noticeable in the Green and Red channels, as shown by the examples in Fig. 7, Fig. 8, respectively.

The next step was to extract the RGB values on a root level, these values were then used to create the necessary features for the machine learning dataset. These values were extracted using two different techniques: pixel intensity and colour clustering using the K-means algorithm across different clusters. The RGB values from these two approaches were compared with an observable difference in the range of -5 to 5 for all three channels. Features were also extracted on two more colour spaces: *HSV* and *LAB*, in order to increase the feature dimensionality space for the flesh-colour modelling task. This involved converting the RGB image into HSV and LAB respectively using computer vision techniques.

#### 2.5.1 Flesh-colour prediction modelling

As described in section 2.2, the trained sensory panel rated the sweetpotato root flesh-colour intensity using a scale of discrete values which lie in the range of **0-10**. The flesh-colour prediction problem was modelled as both a regression and classification task. With the regression approach, the *mean orange flesh-colour intensity value* was considered as the label. While for the classification approach, the *mode value* from the ground truth data on the flesh-colour intensity attribute was considered as the label. For both tasks, the input features for the models included the RGB values. The dataset was split 80%:20% for train and test datasets respectively. The training dataset had **89** image samples.

A number of existing classification and regression algorithms were used to perform flesh-colour modelling on the sweetpotato root dataset. For the classification task, experiments were run for two classification models: Decision Tree (DT) and Random Forest classifiers (RF). These algorithms were selected because of their ability to handle large datasets and their ability to minimize overfitting [60] [61]. Classification models were evaluated using *precision* which is the proportion of true positives among the positive predictions [62], *recall* which is the proportion of the correct predictions among all predictions [63] and the *F1-score* which is defined as the harmonic mean of precision and recall [62]. For the regression task, experiments were run on five regression models which included: Linear Regressor (LR), K-Nearest Neighbours (KNN), Decision Tree (DT), Support Vector Machines (SV) and Random Forest Regressors (RF). The Linear Regressor and K-Nearest Neighbors algorithms were selected due to their ability to determine the strength of the features (predictors) in the data [64] while Support Vector Machines was selected because of its ability to handle both linearly and non-linearly separable data [60]. Table 4 shows the main parameters for the algorithms during flesh-colour model training.

**Table 4:** Main parameters considered during flesh-colour model training.

Model	Main Parameters
Linear Regressor	fit_intercept = True
K-Nearest Neighbors	number of neighbours = 5, weights=uniform, metric = minkowski
Decision Tree	criterion = squared error, splitter= best, maximum depth = None, min_samples_split = 2
Support Vector Machines	C=1.0, kernel=rbf, degree =3, gamma =scale
Random Forest Regressor	number of estimators = 100, criterion= squared error, maximum depth = None, min_samples_leaf = 1, max_features = 1

The regression models were evaluated using the *mean absolute error* (MAE), *mean squared error* (MSE) and *R-Squared score* ($R^{2}$). MAE and MSE are common metrics used to measure the error in regression models [64], [65], [66], [67]. The $R^{2}$ score as in machine learning is a correlation between the ground truth and the model predictions [68] and is used to measure the goodness-of-fit or best-fit line of a regressor model to the ground truth.

### 2.6 Exploratory data analysis for mealiness modelling

As discussed in section 2.4, after the background extraction process the next step was the exploratory data analysis to understand the features that could be used to develop the mealiness prediction models.

***RQ1: What are the most important image features that contribute to the sweetpotato root flesh-colour and mealiness attributes?***

**Fig. 9:**
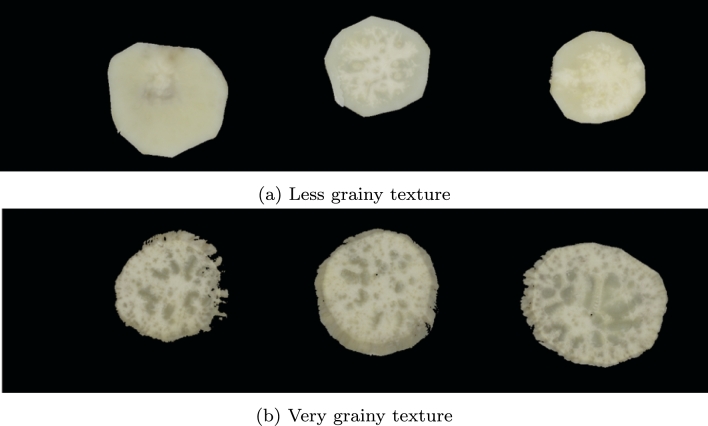
Sample sweetpotato root images showing different degrees of graininess.

The mealiness prediction was modelled as a regression problem where the input data was the *sweetpotato images* and the target was the mean *mealiness by hand* value given by the trained sensory panel in Table 1. The task here was identifying features in the input images to predict the mean *mealiness by hand* value. We considered manual and automated feature extraction procedures as possible approaches for mealiness modelling. For the manual feature extraction process, we focused on identifying features which could be related to the texture of the boiled sweetpotato root. The texture as felt by hand is one of the indicators used by the sensory panel experts to evaluate mealiness [16]. From an image, the texture can be visually related to how smooth or grainy the surface of the root looks. As shown in Fig. 9, we can observe that the graininess of a root is the perceived uniformity in the texture of a boiled root cross-section image. Although the visual perception of graininess is difficult to quantify, the general assumption is that the more grainy the sample, the more mealy the sample may be.

**Fig. 10:**
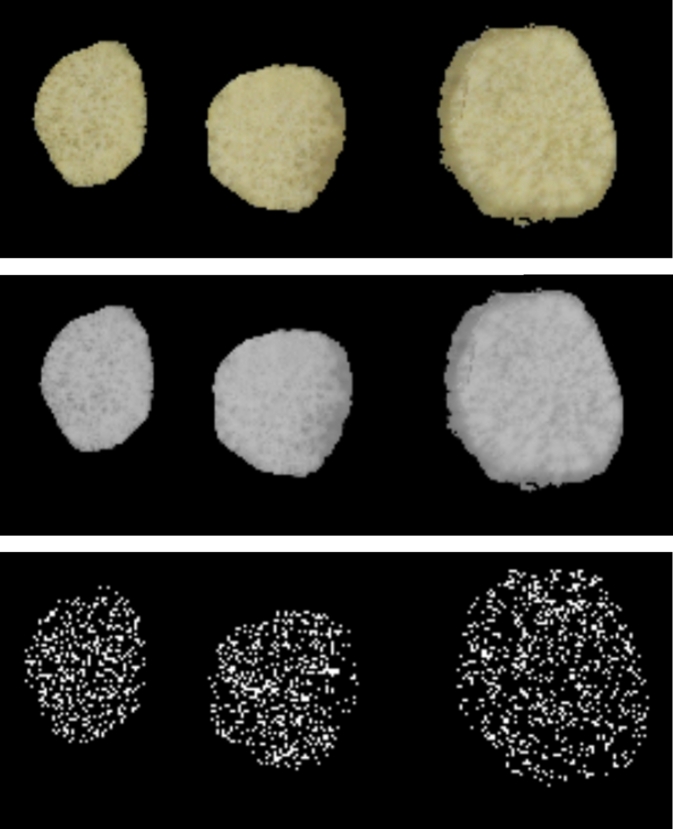
Process for detecting edges in the images from input images to the detected edges in the bottom image.

Computer vision techniques were used to give a measure of the texture of the root surface in the image. This was used to estimate the graininess of an image sample. As a first step, it was necessary to detect contours in the root image. We used the contour detection approach to identify regions in the images where there are differences in pixel values which is a good approximation of the different textures in the images. The second step involved using edge detection approach which identified the boundaries where there were differences between pixels in the image as shown in Fig. 10. Both methods were implemented using OpenCV [59].

Although these methods can help to quantify the texture of the surface of the root cross-sections in the images, a major shortfall is that many of the parameters used to fine-tune the detection are manually configured based on the image characteristics such as flesh-colour. Therefore, it was not possible to have a predefined baseline set of parameters for detecting edges in all the images. For example, for the same image, they may be significant variation in the edge expression just by slightly changing any of the model hyper-parameters. Another issue is that for methods like edge detection, the measure of the graininess of an image would be the number of connected components. This may not be a sound method due to differences in the formation of the edges based on the parameters of detection used. Due to these challenges, we explored an alternative approach to feature identification which could be fairly applied to all the images.

The automated feature selection was done using Convolutional Neural Networks (CNNs) which have shown outstanding capability in modelling features for imaging tasks [69]. For this, we used pre-trained CNNs to analyze and extract features from the convolutional layers just before they are passed to the output layer. Specifically, we used a pre-trained MobilenetV3 [70] model as a baseline and a small model size Efficient-net [71] as a state-of-the-art model. Both models were pre-trained on the ImageNet [72] dataset and could be used to extract base features directly from the sweetpotato root image dataset.

#### 2.6.1 Mealiness prediction modelling

The *mealiness by hand* prediction task was formulated as a regression problem with two target variables: mean *mealiness by hand* and the *positive force 1R*. The main target variable was the mean *mealiness by hand* value as this is what is directly captured by the human sensory panel (Table 1). The *positive force 1R* was selected to explore whether it can be used to approximate mealiness using the same image features. This was based on the assumption that the *positive force 1R* value indicates root firmness which can be indirectly related to the mealiness of a sweetpotato. The dataset used for the experiments contained **127** sweetpotato root images and the corresponding ground truth values of *mealiness by hand* and the *positive force 1R* values. The dataset was split 80%:20% for train and test datasets respectively.

The image test dataset included data from known mealy and non-mealy sweetpotato varieties as specified by the agricultural experts. This was based on information captured from previous harvest trials. The mealy varieties were identified as varieties such as: *New Kawogo, Wagabolige, S36, SPK004, Tanzania, NAROSPOT 1, D20, Silk Omuyaka* that consistently had a *mealiness by hand* score above 4 while the non-mealy varieties such as: *Resisto, Beauregard, NASPOT 9, NASPOT 10, NKB 3, NKB 105, 1.44, Irene* consistently had a *mealiness by hand* score less than 4. The remainder of the test set was randomly selected from the dataset.

Using the extracted features from the CNNs, four machine learning models, i.e., Linear Regression (LR), Random Forest Regression (RF), Gradient Boosting with XGBoost (GR) and a custom Neural Network (NN), were trained to predict the *mealiness by hand* and the *positive force 1R* values. The model selection contained a mix of traditional and state-of-the-art machine learning models. The Linear Regression model was selected as a baseline to compare to the other models. Random Forest Regression was used to evaluate the performance of a tree-based algorithm for the same task. The Gradient Boosting model was used to improve tree-based models like the RF model. The neural network model, a custom model, was developed with five layers (3 hidden). The train set (80%) was used with cross-validation with **5** folds to train the LR, GR and RF models while for the NN, the train set was split 90% and 10% for training and validation, respectively. All the models were tested using the test set (20%).

The LR, RF, and GR models were implemented with *Scikit-learn* [73], a Python programming module for developing machine learning models, while the NN was implemented with *Tensorflow-keras* [74] a python module for developing neural networks. Table 5 shows the main hyper-parameters used for training the RF, GR and NN while the LR model was trained with the default *Scikit-learn* [73] setup. The model evaluation was done using two metrics; Mean Absolute Error (MAE) and the R-Squared ($R^{2}$) score (coefficient of determination). For these experiments, the goal was to obtain model predictions to at least 80% of the *mealiness by hand* human score and the *positive force 1R* for the models to be considered acceptable in practice.

**Table 5:** Model Parameters for training the machine learning models.

Model	Parameters
Random Forest Regressor	maximum depth = 8, number of estimators = 20
Gradient Boosting	learning rate = 0.1, max depth = 8, number of estimators = 200
Neural Network	optimizer = adam, learning rate = 0.0001, loss = MSE, epochs = 5000,
	Early stopping (if the validation MAE does not reduce by more than 0.005 for 100 epochs). The model was trained for 1664 epochs.

## 3 Model results and evaluation

After the model development, the next step carried out was the model evaluation. The results in this section were driven by answering the second research question.

***RQ2: How do different machine learning algorithms perform in predicting flesh-colour and mealiness attributes of sweetpotato roots using image analysis?***

### 3.1 Flesh-colour model results

Table 6, Table 7 show the summary for the scores of the best-performing models on the regression and classification flesh-colour modelling tasks using the standard machine learning evaluation metrics. Table 6 shows that the Linear Regression model was the best-performing regression model with an $R^{2}$ value of 0.92 and an MSE of 0.58. The random forest classifier was the best-performing classifier model with a precision of 0.73, Recall of 0.64 and F1-score of 0.67 as depicted in Table 7. However, the regression models performed better than the classification models, given that we had an imbalanced dataset across the different classes. The flesh-colour classes included: white, cream, yellow, yellow-orange, orange, and deep orange, which were coded as 0,1,3,5,8 and 10, respectively. 70% of the samples in the dataset were in the 0-5 range.

**Table 6:** Flesh-colour prediction results from the regression models.

Model	*R*^2^	MSE
Decision Trees Regressor	0.75	1.86
Linear Regressor	**0.92**	**0.58**
Random Forest Regressor	0.87	0.67

**Table 7:** Flesh-colour prediction results from the classification models.

Model	Precision	Recall	F1-Score
Decision Tree Classifier	0.70	0.50	0.56
Random Forest Classifier	**0.73**	**0.64**	**0.67**

***RQ3: How do machine learning model results for flesh-colour and mealiness sweetpotato root sensory attributes compare to the trained sensory panel flesh-colour and mealiness scores?***

To better assess the results from the regression models, we performed error analysis on the sweetpotato image test dataset. This included test samples used to compare the results from the best regression model with the ground truth data values for the mean *Orange flesh-colour Intensity* as shown in Table 1. From this analysis, we deduced that the flesh-colour regressor model was learning some relevant information from the features and thus it is able to relate the extracted features to the mean *orange flesh-colour intensity* value which is the label or output for the regressor model within an acceptable error rate in the range of the trained sensory panel score. Table 8 shows the results of the orange flesh-colour intensity values for selected genotypes. The values include scores from the model predictions, scores given by the sensory panel, and the absolute error showing the difference between the ground truth and the predicted values.

**Table 8:** Comparison of the predicted flesh-colour model results and ground truth results from the trained sensory panel.

Variety Name	Predicted	Ground	Absolute
	Value	truth	Error
C_P_UGP20170334-27 R3	0.886	0.400	0.486
C_P_SILK OMUYAKA R3	1.405	0.311	1.094
C_C_UGP20170335-6 R2	2.738	1.545	1.193
C_P_UGP20170028-7 R1	4.879	3.909	0.970
C_C_UGP20170015-26 R2	0.492	1.400	0.908
C_C_EJUMULA R1	7.472	8.182	0.709

We can consider the results for *C_C_EJUMULA R1* as shown in Table 8, the mean ground truth value given by the human sensory panel was 8.182, while the model prediction score was 7.472 which gave an error rate of 0.709. The results show that the predicted model results are within the acceptable range of ground truth values between 7-9.

### 3.2 Mealiness model results

Table 9, Table 10 show the evaluation results for the machine learning models for *mealiness by hand* and *positive force 1R* respectively. From the results in Table 9, the best-performing model was Random Forest which attained an $R^{2}$ value of 0.85, followed by the XGBoost and Neural Network models with $R^{2}$ scores of 0.80 and 0.77 respectively. Considering the $R^{2}$ value of 0.80 threshold for comparison to the ground truth values from the trained sensory panel, the performance of the Random Forest and XGBoost models was comparable to the ground truth values. Although the Neural Network model had the lowest MAE, the difference in the $R^{2}$ score was considered more significant in the selection of the Random Forest whose MAE was not so high either.

**Table 9:** Mealiness prediction model performance for the *mealiness by hand* sensory attribute.

Model	*R*^2^	MAE
Linear Regressor	0.57	2.30
Random Forest Regressor	**0.85**	0.90
XG Boost	0.80	1.13
Neural Network	0.77	0.81

**Table 10:** Mealiness prediction model performance for the *positive force 1R* attribute.

Model	*R*^2^	MAE
Linear Regressor	0.50	2384
Random Forest Regressor	**0.83**	533
XG Boost	0.76	1473
Neural Network	0.79	971

For the prediction of the *positive force 1R*, the Random Forest had the best scores with an $R^{2}$ score and MAE score of 0.83 and 533 respectively. This was followed by the Neural Network and XGBoost models with $R^{2}$ scores of 0.79 and 0.76 respectively. The performance of both the Random Forest and Neural Network are comparable to the trained sensory panel results based on the $R^{2}$ threshold, and the RF model was chosen for deployment. To better understand the model results, we also performed error analysis on a new image dataset. This included new samples to test and compare the results from the best machine learning model and the ground truth data which is the mean *mealiness by hand* value given by the sensory panel as shown in Table 1.

Further analysis between the Random Forest model which was the best performing model and the trained sensory panel ground truth was carried out to get a better understanding of the differences between the best and worst predictions from the test set. The results from this comparison are shown in Table 11 and they show that the RF model was able to perform with a relatively low error even for the worst 3 predictions except for the variety *BEAUREGARD* which was an outlier with an exceptionally high error. We believe this is due to the fact that this variety had only one sample in the entire image dataset.

**Table 11:** Comparison between the model predictions and the ground truth for the *mealiness by hand* sensory attribute.

Variety	Predicted value	Ground truth	Absolute Error
**Best 3**			

UGP20170907-31	6.667	6.696	0.029
UGP20170341-20	7.333	7.199	0.134
UGP20170342-12	6.000	5.851	0.149

**Worst 3**			

UGP20170334-13	7.111	5.488	1.623
SILK OMUYAKA	7.944	6.154	1.790
BEAUREGARD	0.727	6.376	5.649

## 4 Tool development and deployment

After model development and deployment, the last two steps as shown in Fig. 1 were model deployment and expert training. The deployment was driven by the fourth research question:

***RQ4: Can we develop a tool for breeders to predict flesh-colour and mealiness for sweetpotato samples?***

In order to allow for interaction with the models and for the agricultural experts to run the flesh-colour and mealiness prediction seamlessly, there was a need to have an environment to deploy and ease interaction with the developed models. The tool is a website through which breeders can upload images and it has a python back-end which can either be installed as PC application or remotely on the internet. The experts were aided to carry out multiple experiments on sweetpotato root image samples for extraction of flesh-colour and mealiness sensory attributes at an increased throughput compared to the human sensory panel.

**Fig. 11:**
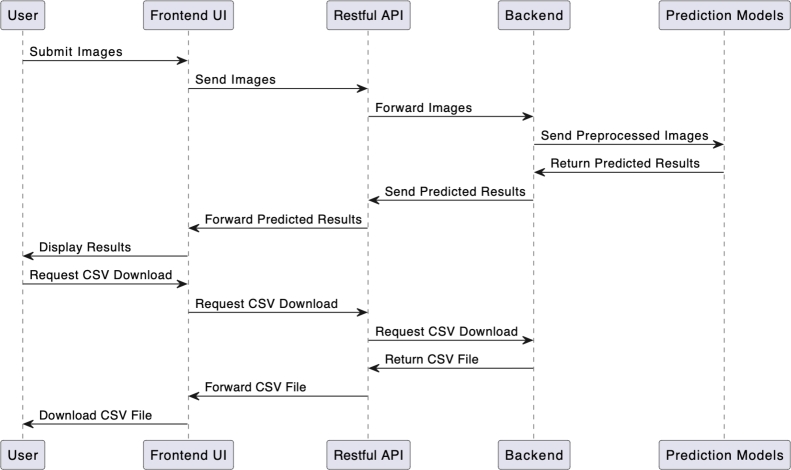
Flow diagram for the sweetpotato root sensory attribute prediction tool.

The overall architecture of the sweetpotato root sensory attribute prediction tool is shown in Fig. 11. The tool takes as input sweetpotato images captured using the DigiEye which are passed to the flesh-colour and mealiness prediction models. The models then predict the flesh-colour and mealiness sensory attributes and return the results of the submitted images. The tool also allows download of the model results in a Comma Separated Value (CSV) file. The tool was developed in a Python programming language using the Django framework.

The tool is composed of three main modules:

• Front-end that allows interaction with the end users.
• Back-end that handles the system logic and the trained sensory prediction models.
• Restful API that bridges the communication between the back-end and front-end.
• Prediction models that provide the colour and mealiness prediction results.

**Fig. 12:**
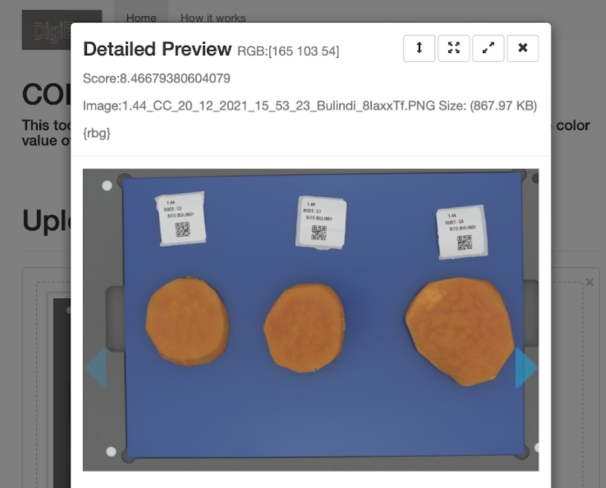
Colour prediction results for variety “Bulindi_8” with an RGB flesh-colour score of 165,103,54 and an orange intensity score of 8.467.

**Fig. 13:**
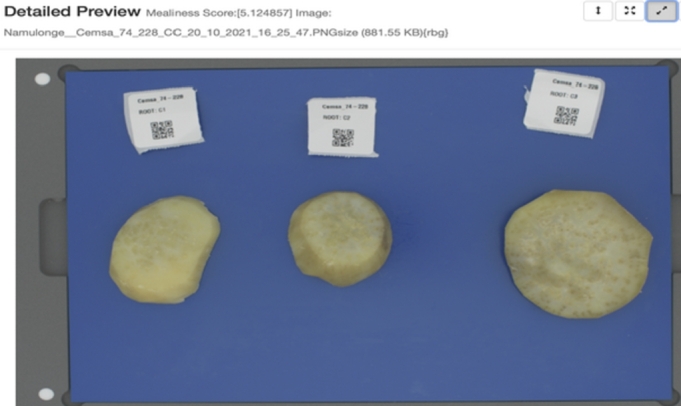
Mealiness prediction results for the variety “Namulonge_Cemsa 74-228 CC” with a mealiness score of 5.124.

A user loads the user interface as a web page in a browser and chooses to either use the flesh-colour or mealiness prediction components. The user then proceeds to upload images captured with the DigiEye machine through the browser and submit them for analysis. The images are processed in the back-end and predicted values are sent back to the user interface and rendered to the user as shown in Fig. 12, Fig. 13 showing the preview of the uploaded image, the image name, flesh-colour with the extracted RGB values or mealiness prediction score and the image size. The detailed steps on the use of the prediction tool are highlighted in the standard operating procedures for image capture in sweetpotato root sensory attribute prediction [47].

After the model deployment, the research team participated in a tool transfer session to CIP-Uganda team. This included the deployment of the software prototype for the flesh-colour and mealiness prediction models, training of breeders on how to use the tool and demonstrating how the sensory attribute prediction tool can be integrated into their current sensory attribute screening process. Time measurements were taken to assess how long it took for the tool to return predictions after images were uploaded. These measurements were captured as an average of three runs (a run measures the time taken from when images are uploaded to when the results are displayed to the user) of 50 images each for flesh-colour and mealiness prediction and the results of this analysis as shown in Table 12.

**Table 12:** Summary of time measurements for different runs for mealiness and flesh-colour prediction with the sensory attribute prediction tool.

**Run**	**Total Time taken (seconds)**	**Average per image**
**Flesh-colour prediction**		

1	111	2.22
2	105	2.1
3	99	1.98

**Mealiness prediction**		

1	301.6	6.03
2	276	5.52
3	268	5.36

## 5 Discussion

The results show that machine learning techniques can be used for the prediction of flesh-colour and mealiness sweetpotato sensory attributes based on root image data. Whereas the flesh-colour measurement task could be modelled both as a classification and regression task, from this study we have seen that the regression models performed better than the classification models. This could be a result of the class imbalance problem, which is an important factor when developing classification models while it does not affect the regression models adversely. The majority of the samples in the machine learning dataset belonged to classes: white, cream, yellow and yellow-orange which were coded as 0, 1, 3 and 5 respectively, in the ground truth data. Nonetheless, it is clear from the experiments conducted for both tasks that pixel intensity values extracted using computer vision techniques can be used as input features to machine learning models can be used to understand the various flesh-colour variations. The outcomes of the regression models also demonstrate that the flesh-colour measurement task in sweetpotato root can be automated with a tolerable level of accuracy. This is demonstrated by the linear regression model's $R^{2}$ value of **0.92**, which is higher than the cutoff of **0.80**. The results obtained are within the acceptable ranges of the ground truth values given by the trained sensory panel.

From the results obtained in the *mealiness by hand* experiments, it is evident that computer vision techniques can be used to predict the mealiness of boiled sweetpotato root images. Three of the models used that is Random Forest, XGBoost and Neural Network algorithms are able to predict the *mealiness by hand* attribute values all scoring $R^{2}$ values above **0.75** on the test set. This shows that these techniques can be used to automate the mealiness scoring task with high accuracy. Specifically, the Random Forest and XGBoost algorithms scored an $R^{2}$ value of 0.85 and 0.80 respectively which matched the **0.80** threshold for comparability to the trained sensory panel results. This implies that root flesh-colour and mealiness can be predicted by analyzing high-quality DigiEye images of boiled sweetpotato roots with a relatively high precision.

The sweetpotato root sensory attribute prediction tool prototype demonstrates that the mealiness and flesh-colour prediction models can be easily deployed for use by the breeders. This tool provides results in a format that can be easily integrated in the sweet potato breeding pipeline which eliminates the need to develop special methods to interpret the results. The potential to deploy this tool as a web service means that it can be used widely as long as one has access to the internet and a web-browser.

The work provides sufficient evidence that imaged-based prediction of flesh-colour and mealiness sweetpotato sensory with machine learning can be done with good accuracy. Based on the time taken to analyze an image using the tools shown in Table 12 combined with the time required to prepare and capture an image with the DigiEye system (1 minute), the proposed solution requires approximately only 5% of the reported time taken by a human expert (30 minutes) on the sensory panel to give results. Although it should be noted that the sensory panelist scores up to 22 other attributes during this time. Therefore, the models can be deployed in versatile tools which can be used by the breeders to significantly increase throughput during the screening of flesh-colour and mealiness sensory attributes.

There are several limitations to the research work. The first weakness is the dataset size. We report using 217 sweet potato samples as described in Section 2. However, most of these samples were categorized under white, cream-yellow and yellow-orange colour classes leading to a data imbalance problem. As a result, modelling the problem as a classification task did not yield good results, limiting the applicability of classification for flesh-colour prediction.

The trained sensory panel provided the ground truth data used in training and evaluating the machine learning models, as described in Section 2. However, conducting sensory panels is time-consuming and as a result only 227 ground truth data samples were obtained and used in this research. This limited the size of the ground truth dataset used during model training for flesh-colour and mealiness prediction.

The adaptation of machine learning models to new domains requires training with domain-specific data. For example, the models built for flesh-colour and mealiness prediction in Uganda might not work well when applied to data from a different region like Mozambique. This could be due to differences in the flesh-colour scale, mealiness scale or the phenotypic characteristics of the varieties grown in these regions [12]. The process for implementing the proposed solution might require the collection of new datasets and re-training of the models. As a result, it is important to collect training data for these environments to have models that can provide acceptable predictions for given sensory attributes.

## 6 Conclusion

In this paper, we have presented the application of machine learning techniques to analyze high-quality DigiEye images of sweetpotato roots to measure two main sensory quality attributes: flesh-colour and mealiness. We built machine learning models for flesh-colour and mealiness prediction on image samples of boiled sweetpotato roots. The models were deployed and tested by agricultural experts on sweetpotato samples. Our results show that we can use machine learning with image-based analysis techniques to automate the prediction of flesh-colour and mealiness of boiled sweetpotato root tubers with high accuracy. As part of future work, we want to improve the machine learning models to predict other sweetpotato flesh-colour variations like purple-fleshed sweetpotato root varieties at the International Potato Center in Mozambique. Furthermore, we will focus on building image-based models for predicting other sensory attributes like water absorption, brownness, fibrousness and firmness, which are important sweeetpotato sensory attributes. The developed image-based analysis tools will also be available to the broader sweetpotato breeding community through the model integration within the Breedbase ecosystem.

## Declaration of Competing Interest

The authors declare that they have no known competing financial interests or personal relationships that could have appeared to influence the work reported in this paper.

## Data Availability

Data will be made available on request.

## Footnotes

1 https://www.makesense.ai/.
